# Management of Gestational Gigantomastia with Goldilocks Procedure after Mastectomy: A Case Report and Review of Literature

**DOI:** 10.1055/a-2181-8621

**Published:** 2024-02-07

**Authors:** Ho Yoon Jeong, Taewoo Kang, Heeseung Park, Kyoung Eun Kim, Su Bong Nam, Ju Young Go, Seong Hwan Bae

**Affiliations:** 1 Department of Plastic and Reconstructive Surgery, Pusan National University School of Medicine, Busan, Republic of Korea; 2 Busan Cancer Center and Biomedical Research Institute, Pusan National University Hospital, and Department of Surgery, Pusan National University, School of Medicine, Busan, Republic of Korea; 3 Biomedical Research Institute, Pusan National University Hospital, Busan, Republic of Korea; 4 Department of Plastic and Reconstructive Surgery, Pusan National University School of Medicine, Yangsan, Republic of Korea; 5 Atelier Plastic Surgery Clinic, Seoul, Republic of Korea

**Keywords:** gigantomastia, mastectomy, breast

## Abstract

Gestational gigantomastia is characterized by the rapid growth of breasts during pregnancy. The treatment method of gestational gigantomastia is unclear; if the medical treatment is ineffective, surgery is considered. However, sufficient research on which method is best to perform breast reconstruction for the gestational gigantomastia patient has not yet been conducted. Our patient was young and had aesthetic needs; thus, we did not recommend modified radical mastectomy. However, it was difficult for the patient to consider active reconstruction using an implant or autologous tissue because of the expected complications and economic problems. The patient had a thin body shape and very large breasts compared with the trunk. Therefore, breast volume was not significantly required after reconstruction. Additionally, we expected that a considerable portion of skin would remain after mastectomy as a tubular-shaped breast. It was expected that the Goldilocks technique would be sufficient to meet the patient's volume needs. Therefore, we proceeded with total mastectomy and reconstruction using the Goldilocks procedure. No complications were recorded after the operation; most of the patient's discomfort was resolved, and the shape and size of the breasts were satisfactory.

## Introduction

Gestational gigantomastia is a rare disease, and its definition, etiology, and treatment methods have not been established. It is characterized by rapid and disproportionate enlargement of the breasts during pregnancy 1 or an enlargement of the breast, wherein >1,500 g of breast tissue should be removed from the breast. 2 It occurs with a prevalence of approximately 1/28,000 to 1/100,000 during pregnancy. 3 It is prevalent in multiparous women than in first-time pregnant women. 3 4

Moreover, the treatment method of gestational gigantomastia is unclear, and if the medical treatment is ineffective, surgery should be considered. After total mastectomy, breast reconstruction is considered in various ways; however, sufficient research on which method is best to perform breast reconstruction for the gestational gigantomastia patient has not yet been conducted. We report a patient with severe bilateral gestational gigantomastia, and a good result after total mastectomy and reconstruction through the Goldilocks procedure.

## Case

The patient was a 36-year-old primipara (gravidity, preterm, abortion, living (GPAL) 0-0-0-0) with no history of contraception. She had no specific underlying disease or family history and had a regular menstrual cycle of 30 days. She usually experienced a change in breast size according to her menstrual cycle with no breast pain. She was administered tamoxifen for 2 to 3 months before pregnancy because of the development of new nodules, breast swelling, and breast pain.

Before pregnancy, her height and weight were 165 cm and 64 kg, respectively, her body mass index (BMI) was 23.5, and her brassiere size was 80D. She reported that she felt her breasts had gradually become heavier from week 6 of pregnancy.

At the first visit to our hospital in October 2021, both breasts had chronic skin changes, ulcerative lesions, and skin heat. The breasts were considerably large to extend to the pelvis, and bilateral huge axillary accessory breasts were observed. During the follow-up period of the pregnancy, a gradual increase in breast size was observed as the gestational age advanced.

No fetal problems were recorded during pregnancy. However, uterine contractions and a short cervix were observed in intrauterine pregnancy at 36 weeks, and an elective cesarean section was performed at 36 ^+2^ weeks. The fetus was 2.35 kg with an Apgar score of 8 and 9 at 1 and 5 minutes, respectively. The fetus was a normal infant without remarkable findings.

After the completion of pregnancy, her weight was 70 kg (BMI, 25.7), and cavelactin and tamoxifen were administered for 3 weeks to reduce the breast volume. After medication, the breast size temporarily decreased; however, it was insignificant. Additionally, the shoulder and back pain due to the heavy and large breasts and discomfort, which prevented her from performing daily life activities, continued. Therefore, we decided to perform the surgery. Before surgery, the patient underwent full laboratory investigations, including complete blood count, liver function tests, renal function tests, hormonal assays (estrogen, progesterone, prolactin, testosterone, follicle-stimulating hormone, luteinizing hormone, thyroid-stimulating hormone, T3, T4, and insulin), immunological assay (anti-double stranded deoxyribo nucleic acid), electrolytes, and lipid tests (low-density lipoprotein-cholesterol, high-density lipoprotein-cholesterol, triglyceride). All test results were within the normal range.

The patient opted for total mastectomy without reconstruction, considering the extent of discomfort and cost of surgery. After sufficient consultation with her, we decided to reconstruct her breasts with the Goldilocks procedure, which was cosmetically satisfactory compared with total excision and relatively inexpensive.

**Fig. 1:**
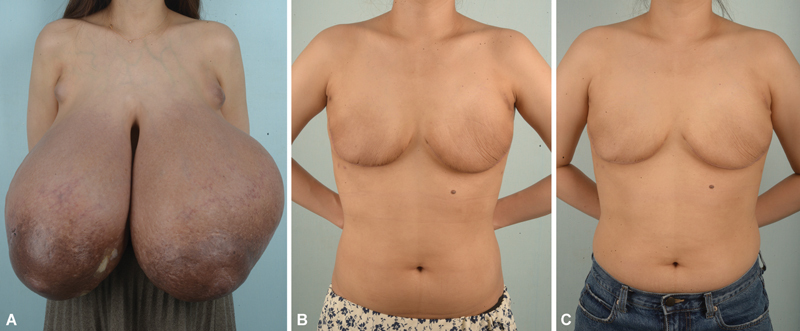
( **A** ) Preoperative frontal view. ( **B** ) Postoperative 3-month frontal view. ( **C** ) Postoperative 6-month frontal view.

After surgery, biopsy results revealed fibroadenomas of various sizes and tubular adenomas with increased benign-looking lymphatics. No complications, such as hematoma, skin flap necrosis, or infection, were encountered. The patient was satisfied that the back and shoulder pain had completely improved, and the restrictions on daily life activities had been mitigated. Her weight was 63 kg (BMI, 23.1) 6 months after the surgery ( Fig. 1 ).

### Surgical Procedure

Becker et al have reported the Goldilocks procedure without a vertical incision. 15 We performed the operation using this method. Skin markings were performed in an upright position before entering the operating room. The inframammary folds were assessed and marked on both sides.

**Fig. 2:**
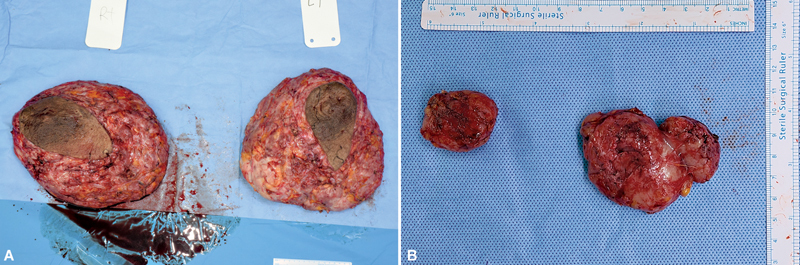
( **A** ) Resected bilateral breast. The removed right and left breasts weighed 3,400 g and 4,100 g, respectively. ( **B** ) Resected bilateral axillary accessory breast. The removed right and left axillary breasts weighed 8.7 and 29.7 g, respectively.

Skin-sparing mastectomy through a periareolar incision was performed to resect the breast tissue after general anesthesia. After bilateral mastectomies, the removed right and left breasts weighed 3,400 and 4,100 g, respectively. Additionally, the bilateral axillary accessory breasts were resected. The removed right and left axillary breasts weighed 8.7 g and 29.7 g, respectively. A permanent biopsy of the resected mass was performed ( Fig. 2 ).

**Fig. 3:**
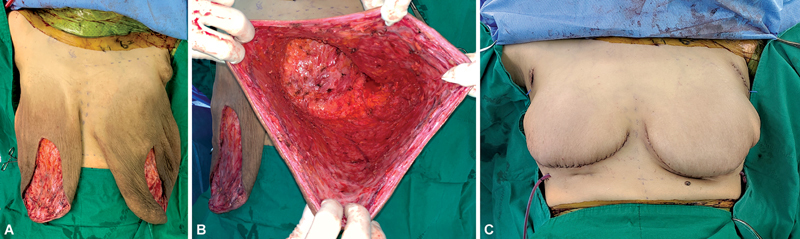
( **A** ) After skin sparing mastectomy, the mastectomy flap was tubularly long. ( **B** ) After skin sparing mastectomy, the mastectomy flap was tubularly long. ( **C** ) Immediate postoperative frontal view, taken in a sitting position.

After surgical design for the Goldilocks procedure, the inferior pole of the breast was deepithelialized, and a dermal flap was created. After deepithelialization, we folded the inferior portion of the dermal flap superiorly and sutured it with Vicryl 2–0 to the muscle fascia for anchoring. The lateral and medial portions of the dermal flap were then folded above the inferior portion of the dermal flap to provide extra projection, and the flap edges were sutured with Vicryl 2–0 at the flap margins. A Jackson–Pratt drain was inserted in the pocket and anchored to the skin. The superior mastectomy flap was advanced inferiorly to cover the dermal flaps and anchored to the inframammary fold to create the breast mound. Skin closure was performed along the inframammary folds ( Fig. 3 ).

## Discussion

Gestational gigantomastia is a rare disease, the etiology of which is unknown, and its treatment methods are not standardized. Because it is a pregnancy-related disease, termination of pregnancy has been discussed. However, currently, it is no longer recommended for ethical or therapeutic purposes. 4 In exceptional cases where the pregnant woman's life is at risk, it may be recommended to consider termination of the pregnancy or induction of preterm labor. 5

Hormonal excess, elevated prolactin levels, and autoimmune disorders are correlated. Increased estrogen levels during pregnancy contribute to gestational gigantomastia, potentially treated with tamoxifen, although its efficacy is uncertain. 5 6 Bromocriptine is used to reduce prolactin levels and can stabilize the condition until the end of pregnancy, without increasing the risk of miscarriage or birth defects. 4 The effectiveness of nonsteroidal anti-inflammatory drugs or corticosteroids for autoimmune-related cases remains inconclusive. 5 7

Medical treatments may yield temporary effects but are unlikely to restore the breasts to their original size, 7 particularly in cases where the patient is within the normal range upon evaluation, limiting the choice of medical treatment. As a result, surgical treatment is often chosen in most cases. Patients have the option to choose between breast reduction or mastectomy. Breast reduction provides the advantage of preserving the ability to breastfeed; however, there is a higher chance of recurrence during future pregnancies. 5 7

We examined several articles about breast reconstruction methods following the removal of gestational gigantomastia before the operation. However, previous studies on gestational gigantomastia have mostly focused on the removal of breast tissue rather than reconstruction methods. 8 9 10 11 12 In particular, many studies discuss the pros and cons of choosing between simple mastectomy and breast reduction. According to the literature review by Shoma et al, among 46 articles, there were an equal number of cases, 16 each, where mastectomy and reduction mammoplasty were performed. 6 For the reconstruction methods following simple mastectomy, the majority involved delayed reconstruction using tissue expanders or direct-to-implant reconstruction. 7 13 14 In the case of delayed reconstruction, the use of the deep inferior epigastric artery perforator flap or implant exchange has been reported. 3 There have also been reports of reconstruction using the latissimus dorsi muscle flap immediately after mastectomy, but finding reports on other reconstruction methods was extremely difficult. 6

In this study, it was difficult for the patient to consider active reconstruction using an implant or tissue expander because of the expected complications and economic problems caused by excessively large breasts. However, the patient was young and had aesthetic needs; hence, we did not recommend modified radical mastectomy. Moreover, implant and autologous reconstruction were not considered because of cost and donor site morbidity. The patient had a thin body shape and very large breasts compared with the trunk. Therefore, breast volume was not significantly required after reconstruction. Additionally, we expected that a considerable portion of skin would remain after mastectomy as a tubular-shaped breast. It was expected that the Goldilocks technique would be sufficient to meet the patient's volume needs.

After skin-sparing mastectomy, it was determined that vertical halving incision would be detrimental to flap survival because the mastectomy flap was tubularly long. Therefore, we referred to Becker et al's Goldilocks procedure without a vertical incision rather than the traditional wise pattern of Goldilocks. 15 Various surgical methods have been used to treat gigantomastia. However, there have been no reports of the use of the Goldilocks procedure.

The Goldilocks procedure, published in 2012, is frequently offered as a single-stage operation for patients with high BMIs and medical comorbidities that render them poor candidates for more complex options, including implant-based and autologous breast reconstruction. 16 The Goldilocks procedure has fewer complications and a shorter operating time. 16 17 Additionally, it can be selected as the first choice for single-stage operation of reduction in obese patients or patients with large breasts. 18 It is a safe bridge without complications even if a two-stage reconstruction is planned. 16 19

According to Manrique et al, 16 the overall complication rate after mastectomy with the Goldilocks procedure remained in the range of 8 to 9.4%, similar to this study. It is difficult to create a breast volume that satisfies the patient's aesthetic appearance with only the Goldilocks procedure, and the rate of additional operation to correct it is approximately 40.7%, of which 37% was corrected through a fat graft. We observed a greater change in breast volume due to postoperative absorption. Furthermore, the patient was satisfied with the shape of the breast after surgery. No other complications were noted. However, relatively a short follow-up period of 6 months has limitations in evaluating the success of the Goldilocks procedure.

In conclusion, we experienced a case of severe gestational gigantomastia and obtained a good result after total mastectomy and reconstruction using the Goldilocks procedure. After surgery, most of the discomfort caused by the large breasts disappeared completely, and the shape and size of the breasts were satisfactory. This is the first case of reconstruction using the Goldilocks procedure after mastectomy in a patient with gestational gigantomastia. Therefore, the Goldilocks procedure may be a good reconstruction option for gestational gigantomastia.
